# Homogeneous Electrochemical Aptamer Sensor Based on Two-Dimensional Nanocomposite Probe and Nanochannel Modified Electrode for Sensitive Detection of Carcinoembryonic Antigen

**DOI:** 10.3390/molecules28135186

**Published:** 2023-07-03

**Authors:** Zhengzheng Yan, Shiyue Zhang, Jiyang Liu, Jun Xing

**Affiliations:** 1 General Surgery Department, Shanxi Bethune Hospital, Shanxi Academy of Medical Sciences, Tongji Shanxi Hospital, Third Hospital of Shanxi Medical University, Taiyuan 030032, China; yanzhengzheng@sxmu.edu.cn; 2 Department of Chemistry, Zhejiang Sci-Tech University, Hangzhou 310018, China; 202120104186@mails.zstu.edu.cn; 3 Department of Breast Surgery, Shanxi Bethune Hospital, Shanxi Academy of Medical Sciences, Tongji Shanxi Hospital, Third Hospital of Shanxi Medical University, Taiyuan 030032, China

**Keywords:** electrochemical aptamer sensor, homogeneous, two-dimensional nanocomposite probe, nanochannel array, carcinoembryonic antigen

## Abstract

A rapid and convenient homogeneous aptamer sensor with high sensitivity is highly desirable for the electrochemical detection of tumor biomarkers. In this work, a homogeneous electrochemical aptamer sensor is demonstrated based on a two-dimensional (2D) nanocomposite probe and nanochannel modified electrode, which can realize sensitive detection of carcinoembryonic antigen (CEA). Using π-π stacking and electrostatic interaction, CEA aptamer (Apt) and cationic redox probe (hexaammineruthenium(III), Ru(NH_3_)_6_^3+^) are co-loaded on graphite oxide (GO), leading to a 2D nanocomposite probe (Ru(NH_3_)_6_^3+^/Apt@GO). Vertically ordered mesoporous silica-nanochannel film (VMSF) is easily grown on the supporting indium tin oxide (ITO) electrode (VMSF/ITO) using the electrochemical assisted self-assembly (EASA) method within 10 s. The ultrasmall nanochannels of VMSF exhibits electrostatic enrichment towards Ru(NH_3_)_6_^3+^ and size exclusion towards 2D material. When CEA is added in the Ru(NH_3_)_6_^3+^/Apt@GO solution, DNA aptamer recognizes and binds to CEA and Ru(NH_3_)_6_^3+^ releases to the solution, which can be enriched and detected by VMSF/ITO electrodes. Based on this mechanism, CEA can be an electrochemical detection ranging from 60 fg/mL to 100 ng/mL with a limit of detection (LOD) of 14 fg/mL. Detection of CEA in human serum is also realized. The constructed homogeneous detection system does not require the fixation of a recognitive aptamer on the electrode surface or magnetic separation before detection, demonstrating potential applications in rapid, convenient and sensitive electrochemical sensing of tumor biomarkers.

## 1. Introduction

Cancer is a serious threat to human health and life. Early diagnosis and treatment of cancer are important to improve survival rates of patients. Research has proven that the occurrence and development of cancer is closely related to changes in the expression of protein-related genes within cells. Therefore, the detection of tumor-related protein markers plays a crucial role in the clinical analysis field [1]. Of these, carcinoembryonic antigen (CEA) is the most widely used tumor biomarker, because some tissues, such as the large intestine, pancreas, stomach, breast and medullary thyroid, may overexpress CEA after cancerous transformation, leading to an increase in serum CEA levels [2]. Therefore, sensitive detection of CEA in serum plays an important role in the diagnosis and prognosis evaluation of cancer. At present, many technologies have been reported to detect CEA, including enzyme-linked immunosorbent assay [3], chemiluminescence [4], electrochemiluminescence [5,6,7] and electrochemistry [8,9,10], etc. Among them, electrochemical detection has the advantages of fast analysis, simple instrument, low cost, easy integration and miniaturization [11,12,13]. In addition, compared with antibodies used in traditional immunoassay, aptamer, a new type of nucleic acid recognition molecule that has been manually screened with high affinity and specificity for binding to the target, provides new opportunities for tumor biomarker detection owing to the merits of simple preparation, low cost, easy storage, high stability and wide applicability [14,15]. Therefore, the development of convenient and sensitive electrochemical aptamer sensors for detection of CEA in serum is of great significance.

As protein, tumor biomarkers themselves do not possess detectable electrochemical activity. Thus, the detection mechanism lies in the changes of electrochemical signal caused by the specific recognition between the tumor biomarker and the biological ligand [16,17,18,19]. Commonly, heterogeneous and homogeneous modes are involved. In heterogeneous detection, the biological ligand is usually fixed on the electrode surface, leading to the electrode sensing interface [20,21,22]. However, the construction of electrode sensing interface suffers from cumbersome and time-consuming (e.g., overnight) processes, resulting in poor reproducibility. At the same time, the immobilized recognitive ligand has uncontrollable density and direction, which limits further improvement of selectivity and accuracy in electrochemical analysis. Recently, homogeneous electrochemical sensing has attracted great attention as it does not need the immobilization of the bio-recognitive probe, demonstrating advantages of simple operation, rapid response and high specific efficiency [23,24]. However, it is often necessary to separate the complex formed between tumor markers and adaptor before detection (e.g., using magnetic separation) [25]. Therefore, convenient and highly sensitive electrochemical detection of CEA using a homogeneous aptamer sensor without the separation process is highly desirable.

Nanomaterial-based signal amplification has been an effective strategy to improve the sensitivity of electrochemical sensing. As part of this, porous materials have attracted widespread attention due to their high specific surface area, adjustable structure and pore size. In particular, porous silica materials are most widely used owing to the merits of advantages such as simple synthesis, low cost, high biocompatibility and easy derivatization [26,27,28]. Recently, the growth of vertically ordered mesoporous silica-nanochannel film (VMSF) on the electrode surface has become an important method for preparing an electrochemical sensor with high performance [29,30,31]. VMSF is an ultra-thin nanofilm with uniform nanochannel array perpendicular to the supporting electrode. Commonly, VMSF has adjustable film thickness (usually between 20~200 nm), ultrasmall pore size (usually 2–3 nm) and high pore/channel density (up to 75,000 pores/μm^2^) [32]. Up to now, a large area of VMSF (e.g., tens of square centimeters) can be conveniently prepared using the Stöber solution growth method [33,34]. At the same time, VMSF can also be quickly prepared (usually within 10 s) using the electrochemical-assisted self-assembly (EASA) method [35,36]. In addition, the ultrasmall nanochannel array also endows VMSF modified electrode with unique characteristics. Specifically, some electrochemical probes can be concentrated on the electrode surface, and large-sized molecules/substances will be blocked [37,38]. This is due to the size and electrostatic screening ability of VMSF nanochannels at the molecular level, due to the close diameter to Debye length. After the dissociation of silanol groups (Si-OH, p*K*_a_~2) on the surface of VMSF, VMSF has a negatively charged surface, which can significantly enrich common cationic electrochemical probes, leading to remarkably enhanced signal (e.g., more than two orders of magnitude in electrochemiluminescence detection) [39,40,41]. At the same time, VMSF can block large molecules, particles and two-dimensional (2D) nanomaterials in complex matrices. When constructing functional probes using 2D nanomaterials for homogeneous analysis, for instance, the nanochannels of VMSF can block 2D material and only allow small molecule probes to enter the pores. Therefore, VMSF-modified electrodes can effectively separate or enrich substances with different sizes and charge properties in a complex matrix, achieving the integration of separation, enrichment and detection, demonstrating great potential in direct homogeneous analysis without pre-separation.

In this work, a homogeneous electrochemical sensor for highly sensitive electrochemical detection of CEA is fabricated based on the combination of VMSF electrode with aptamer and redox probe modified graphite oxide nanocomposite. Using π-π stacking and electrostatic interaction, CEA aptamer (Apt) and cationic redox probe (hexaammineruthenium(III), Ru(NH_3_)_6_^3+^) are loaded on graphite oxide (GO), leading to a 2D nanoprobe (Ru(NH_3_)_6_^3+^/Apt@GO). When DNA aptamers recognize and bind to CEA, they dissociate from the surface of GO, leading to the release of Ru(NH_3_)_6_^3+^ probes from the surface of GO to the solution. Based on the electrostatic enrichment of Ru(NH_3_)_6_^3+^ by VMSF and the size exclusion towards GO or Ru(NH_3_)_6_^3+^/Apt@GO, VMSF electrodes can achieve highly sensitive detection of CEA. The constructed homogeneous detection system does not require the fixation of recognitive aptamer on the electrode surface or magnetic separation before detection, demonstrating potential applications in electrochemical sensing of tumor biomarkers.

## 2. Results and Discussion

### 2.1. Fabrication of Homogeneous Electrochemical Sensor for Sensitive Detection of CEA Based on Nanochannel Array Modified Electrode and 2D Nanocomposite Probe

**Figure 1:**
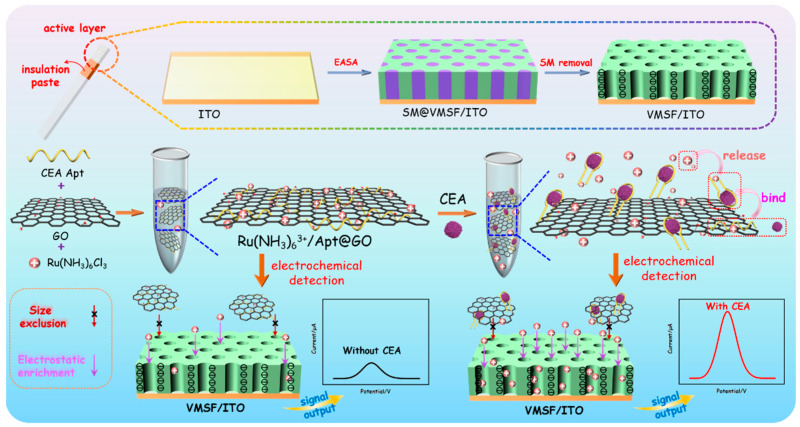
Schematic illustration for the construction of homogeneous aptamer sensor based on a 2D nanocomposite probe and VMSF-modified electrode for sensitive electrochemical detection of CEA.

As shown in Figure 1, an ITO electrode was used as the supporting electrode to grow VMSF through the EASA method, which can realize rapid and convenient growth of VMSF within a few seconds. The mechanism of VMSF growth lies in the hydrolysis and condensation reaction of siloxanes using cationic surfactants (cetyltrimethylammonium bromide, CTAB) micelles (SM) as soft templates. When an ITO electrode is immersed in a tetraethoxysilane (TEOS) solution containing SM and a negative potential is applied, in-situ generated hydroxide ions on the electrode will induce the self-assembly of SM on the electrode surface and catalyze the hydrolysis and condensation of TEOS, leading to nanochannels that are perpendicular to the electrode surface and are filled with SM (SM@VMSF/ITO). After removing CTAB micelles in hydrochloric acid/ethanol solution, open nanochannel array modified electrode (VMSF/ITO) was obtained.

To prepare nanocomposite recognition probes, graphene oxide (GO) with a two-dimensional (2D) structure, excellent water dispersibility and oxygen-containing groups is applied to combine recognitive ligand and electrode probe. DNA aptamer is chosen as the biological molecule to recognize CEA, and the commonly used cationic probe (Ru(bpy)_3_^2+^) is used as redox probe. After the three substances are mixed, Ru(bpy)_3_^2+^/Apt@GO nanocomposite could be easily prepared based on the π-π interaction between single-stranded DNA and GO and the electrostatic adsorption between Apt or GO and Ru(bpy)_3_^2+^. In the presence of CEA, aptamer dissociates from the surface of GO because it recognizes and binds to CEA, while Ru(bpy)_3_^2+^ also releases from the nanocomposite. Free Ru(bpy)_3_^2+^ in the solution can be enriched due to the strong electrostatic effect of VMSF/ITO electrodes, leading to improved electrochemical signal and high detection sensitivity. In addition, the 2D nanocomposite can be excluded by the ultrasmall nanochannels of VMSF, effectively reducing the background signal. As immobilization of recognitive antibodies on the electrode surface and separation before CEA detection are not needed, the constructed homogeneous CEA detection system also has the advantage of simple fabrication and easy operation.

### 2.2. Characterization of VMSF Modified Electrode

**Figure 2:**
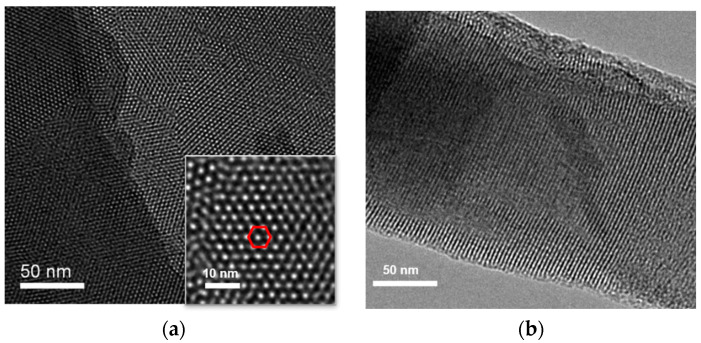
(**a**) Top-view and cross-sectional (**b**) TEM images of VMSF. Inset in (**a**) is the TEM image at high magnificence. The red hexagon indicates that pores are tightly arranged in a hexagonal structure.

The morphology of VMSF grown on the surface of ITO electrode was characterized using transmission electron microscopy (TEM). Figure 2 shows the surface and cross-sectional TEM images of VMSF. As shown in Figure 2a, VMSF has an ordered and uniform pore structure, and the pores are tightly arranged in a hexagonal structure. The pore size of the nanopores ranges from 2 to 3 nm (Figure 2a). The average pore diameter is measured to be 2.6 nm and the pore density is 7.6 × 10^12^/cm^2^, corresponding to a porosity of 40%. Thus, the surface area of the working electrode is estimated to be 0.1 cm^2^ after the removal of SM. The cross-sectional TEM confirms the nanochannel structure (Figure 2b).

**Figure 3:**
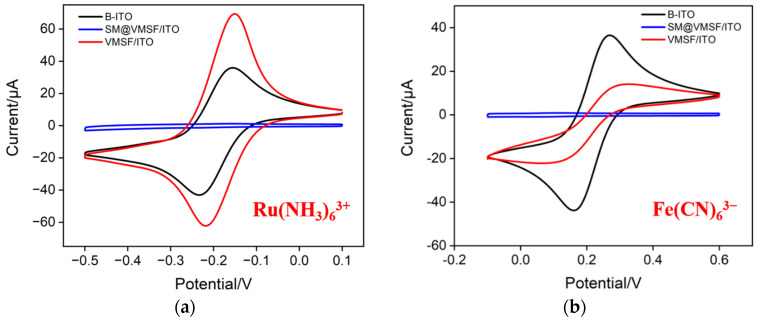
CV curves obtained on bare ITO (B-ITO), SM@VMSF/ITO and VMSF/ITO in KHP (0.05 M, pH = 4) containing 0.5 mM Ru(NH_3_)_6_Cl_3_ (**a**) or 0.5 mM K_3_[Fe(CN)_6_] (**b**).

Using standard electrochemical probes, including negatively charged Fe(CN)_6_^3−^ and positively charged Ru(NH_3_)_6_^3+^ probes, the integrity and charge-selective permeability of VMSF were investigated. Figure 3a,b shows the cyclic voltametric (CV) curves of Ru(NH_3_)_6_^3+^ or Fe(CN)_6_^3−^ obtained on bare ITO, SM@VMSF/ITO and VMSF/ITO, respectively. As can be seen, SM@VMSF/ITO electrode cannot measure the CV signals of the two probes, indicating that the micelles hinder the diffusion of redox probe molecules from the solution to the underlying electrode surface. This phenomenon also proves that VMSF film prepared on the surface of the ITO electrode is intact and crack free. Compared with the bare ITO electrode, the electrochemical signal of the negatively charged Fe(CN)_6_^3−^ probe on the VMSF/ITO electrode reduces. On the contrary, the peak current obtained on VMSF/ITO electrodes enhances in presence of positively charged Ru(NH_3_)_6_^3+^ probes. This is attributed to the negative charge caused by the ionization of silanol groups (p*K*_a_~2) on the surface of VMSF nanochannels. Specifically, negatively charged probes are repelled by VMSF nanochannels, while positively charged probes are attracted, demonstrating the charge-selective permeability of VMSF nanochannels. When Ru(NH_3_)_6_^3+^ is used as a signal probe of homogeneous electrochemical system, the significant enrichment of Ru(NH_3_)_6_^3+^ by nanochannels is beneficial for improving the detection sensitivity.

### 2.3. Characterization of GO and 2D Nanocompostie

**Figure 4:**
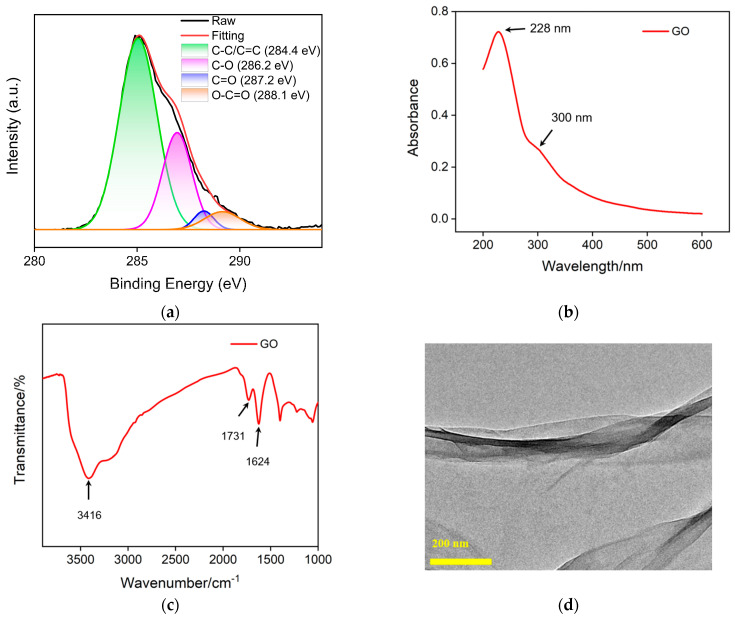
High-resolution C1s XPS (**a**), UV-Vis (**b**) and FT-IR (**c**) spectra of GO. (**d**) TEM image of GO.

Carbon nanomaterials have unique electrical, thermal and optical properties, adjustable structures, high specific surface area, and excellent biocompatibility [42,43,44]. In recent years, nanocarbon materials with different dimensions have received widespread attention, such as 3D graphene [45], 2D graphene or graphene oxide [46], 0D graphene quantum dots [47,48,49], etc. In this work, GO is used as a support carrier for constructing a 2D recognition probe. Figure 4a shows the high-resolution C1s X-ray photoelectron spectroscopy (XPS) spectrum of GO. It can be seen that GO contains four types of carbon bonds, including sp^2^ carbon (C-C/C=C, 284.4 eV), OH/C-O bond (286.2 eV), carbonyl group (C=O, 287.2 eV) and carboxylates (O-C=O, 288.1 eV). Figure 4b displays UV-visible adsorption spectrum of GO. The absorption at 228 nm corresponds to the π-π* transition of sp^2^ carbon, while the absorption band at ~300 nm corresponds to the n-π* transition. Figure 4c shows the FT-IR spectrum of GO, where characteristic peaks can be observed at O-H stretching vibration (3416 cm^−1^), C=O stretching vibration (1731 cm^−1^), sp^2^ carbon (1625 cm^−1^), C-O-C stretching vibration (1300 cm^−1^) and C-O stretching vibration (1050 cm^−1^), proving the existence of oxygen-containing groups on GO. The TEM image in Figure 4d reveals the 2D nanosheet structure.

Zeta potential was measured to investigate the effective preparation of 2D probes. The zeta potential of GO is −73.6 mV, which is attributed to the ionization of oxygen-containing groups on its surface. In the case of aptamer, a zeta potential of −53.8 mV is revealed resulting from its negatively charged phosphate framework. When GO interacts with the aptamer, the zeta potential of Apt@GO is −63.6 mV, indicating that they can bind through π-π interaction. Owint to the electrostatic adsoption of Ru(NH_3_)_6_^3+^, the formed Ru(NH_3_)_6_^3+^/Apt@GO displays a reduced negative charge (−30.2 mV). The phenomenon indicates the successful formation of 2D nanocomposite.

### 2.4. Feasibility of Homogeneous Aptamer Sensing System for Electrochemical Detection of CEA

**Figure 5:**
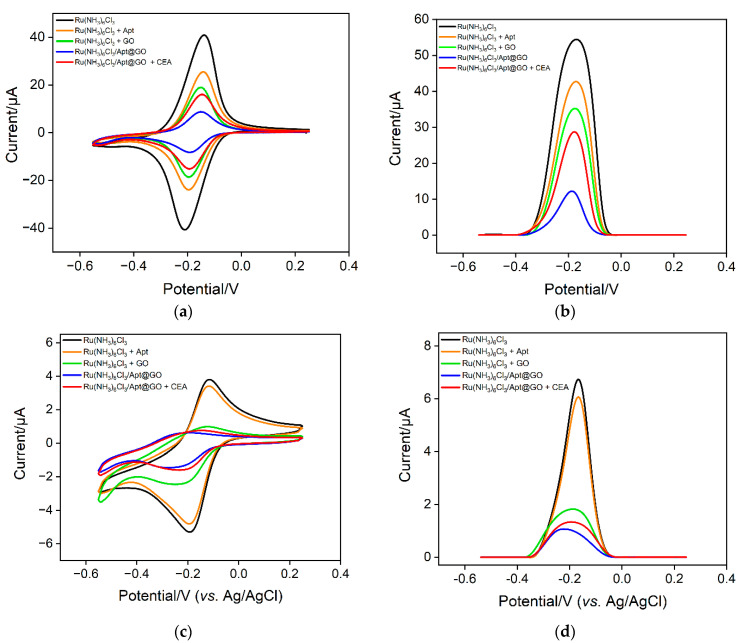
CV (**a**,**c**) and DPV (**b**,**d**) curves obtained on different solution using VMSF/ITO (**a**,**b**) or a bare ITO electrode.

The feasibility of the sensing strategy in this work was verified using two electrochemical methods including CV and DPV. As shown in Figure 5a, when the solution only contains a Ru(NH_3_)_6_^3+^ probe, a pair of reversible redox peaks can be observed on the VMSF/ITO electrode, proving that Ru(NH_3_)_6_^3+^ can freely diffuse through the nanochannels of VMSF to reach the surface of the electrode. When CEA aptamer is added to a Ru(NH_3_)_6_^3+^ solution, the phosphate skeleton of the aptamer can adsorb positively charged Ru(NH_3_)_6_^3+^, resulting in a decrease in the concentration as well as the peak current of Ru(NH_3_)_6_^3+^. When GO coexists with Ru(NH_3_)_6_^3+^, Ru(NH_3_)_6_^3+^ can also be adsorbed because GO contains a large number of oxygen-containing functional groups, which also leads to a decrease in electrochemical signals. In the case of a ternary mixture containing aptamer, GO and Ru(NH_3_)_6_^3+^, the redox signal of Ru(NH_3_)_6_^3+^ significantly reduced because of the formation of nanocomposite and the dual electrostatic adsorption of Ru(NH_3_)_6_^3+^ by both aptamer and GO. When the detection substance, CEA, is present in the solution, the aptamer on the nanocomposite can specifically recognize CEA, leading to the detachment of the aptamer. This also causes the desorption of Ru(NH_3_)_6_^3+^, leading to an increase of electrochemical signals. The same conclusion can be obtained from the DPV measurement (Figure 5b). The above results demonstrate that a homogeneous electrochemical sensor can be constructed based on the nanocomposite probe and nanochannel modified electrode for electrochemical detection of CEA.

To investigate the enrichment of Ru(NH_3_)_6_^3+^ by nanochannels and the improvement of detection sensitivity, the electrochemical signals on ITO in different solutions are investigated. Figure 5c,d shows the CV and DPV signals obtained on a bare ITO electrode. It can be seen that in the Ru(NH_3_)_6_^3+^ solution, the CV or DPV signal on ITO is significantly lower than that obtained on the VMSF/ITO electrode, proving the remarkable enrichment effect of nanochanels on Ru(NH_3_)_6_^3+^. When the bind of CEA on a nanocomposite probe increases the concentration of free Ru(NH_3_)_6_^3+^ in the solution, the electrochemical signal weakly changes. Thus, a bare ITO electrode can not sensitively distinguish the change of increased concentration of Ru(NH_3_)_6_^3+^ in the case of CEA. The above results demonstrate the significant signal amplification effect of nanochannels.

**Figure 6:**
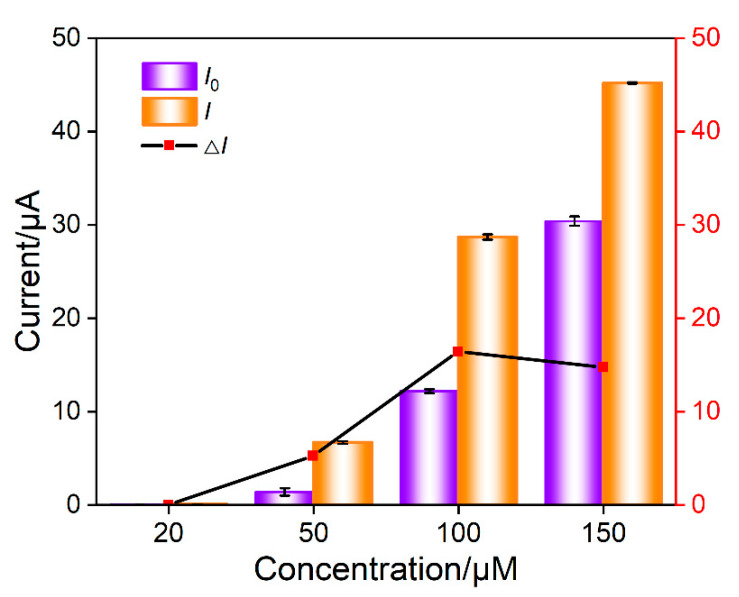
Optimization of concentration of Ru(NH_3_)_6_^3+^ in nanocomposite probe solution. *I*_0_ an *I* are the DPV peak current in absence or presence of CEA, respectively. Δ*I* is the change of DPV peak current (*I* − *I*_0_) after the addition of CEA. The nanocomposite was prepared using GO (0.1 mg/mL) and aptamer (1 μΜ) and different concentration of Ru(NH_3_)_6_^3+^ in PBS (0.01 M, pH = 7.4).

In the mixture of Ru(NH_3_)_6_^3+^ and aptamer GO, the Ru(NH_3_)_6_^3+^/Apt@GO nanocomposite is formed. Although pure nanocomposite dispersions can be obtained by centrifugation or other separation methods, extremely low electrochemical background signals can be obtained due to the exclusion of nanocomposites by nanochannels. However, it is necessary to consider the recovery rate of the separation process, and the storage and dispersion of nanocomposites require careful operations. On the contrary, no separation to obtain pure Ru(NH_3_)_6_^3+^/Apt@GO is performed in this work. Thus, there might be some residual Ru(NH_3_)_6_^3+^. However, GO aptamer and Ru(NH_3_)_6_^3+^ solutions only need to be placed separately. After incubation, freshly prepared nanocomposites can be obtained, making the operation process simpler. The concentration of Ru(NH_3_)_6_^3+^ is optimized to obtain the maximum electrochemical signal change after the addition of CEA. The DPV peak current in the absence (*I*_0_) or presence (*I*) of CEA is measured. Accordingly, the change of DPV peak current (Δ*I* = *I* − *I*_0_) resulting from the addition of CEA is obtained. As shown in Figure 6, the change of DPV peak current is the highest when the concentration of Ru(NH_3_)_6_^3+^ is 50 μM. This has been chosen for further investigation. 

### 2.5. Electrochemical Detection of CEA Using the Fabricated Homogeneous Aptamer Sensor

**Figure 7:**
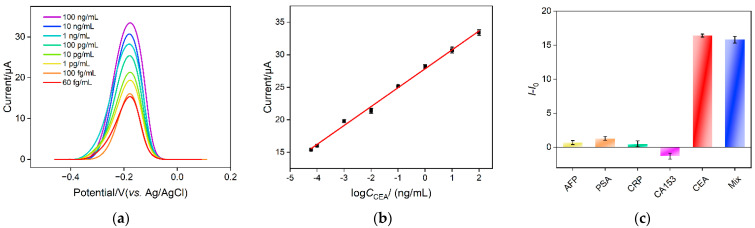
(**a**) DPV curves obtained on nanocomposite probe solution in the presence of different concentrations of CEA. (**b**) The corresponding calibration curve between DPV peak current and logarithmic value of CEA concentration. (**c**) The change of DPV peak current after the addition of different tumor biomarker or their mixture.

Electrochemical detection of CEA is investigated using the developed homogeneous electrochemical sensor based on the combination of a VMSF electrode with 2D nanocomposite recognitive probe. Figure 7a shows the DPV response obtained on a VMSF/ITO electrode when different concentrations of CEA are incubated with the nanocomposite recognitive probe. The detection mechanism lies in the desorption of Ru(NH_3_)_6_^3+^ from the Ru(NH_3_)_6_^3+^/Apt@GO accompanying the binding between aptamer and CEA. As the concentration of free Ru(NH_3_)_6_^3+^ in the solution increases in the presence of CEA, the anodic peak current improves. As displayed in Figure 7b, the anodic peak current (*I*) shows a good linear relationship with the logarithmic value of CEA concentration (log*C*_CEA_) within the range from 60 fg/mL to 100 ng/mL (*I* = 2.91 log*C*_CEA_ + 27.8, *R*^2^ = 0.995). The limit of detection (LOD) is 14 fg/mL, calculated based on a three signal-to-noise ratio (S/N = 3). Table 1 summarizes the comparison between CEA detection performance using different electrodes through electrochemical or ECL detection [5,6,7,8,50,51]. The LOD of the fabricated sensor is lower than that obtained using the sandwich detection mode based on reduced graphene oxide-Pd@Au nanocomposits-thionine-the second antibody/CEA/BSA/the first antibody/chitosan-ferrocenecarboxaldehyde-gold nanospheres modified glassy carbon electrode (rGO-Pd@Au-Thi-Ab_2_/CEA/BSA/Ab_1_/Chi-Fc-Au/GCE) [50], BSA/6-mercapto-1-hexanol/tetrahedral DNA nanostructures/gold electrode (BSA/MCH/TDNs/AuE) [50], gold nanoparticles-bipyridine ruthenium-L-arginine@amino group modified ultrathin Ti_3_C_2_-MXene nanosheets modified GCE (AuNPs- Ru-Arg@NH_2_-Ti_3_C_2_-MXene/GCE) [6] or BSA/the first antibody/glutaraldehyde/chitosan/ionic liquid-platinum compound modified GCE (BSA/Ab_1_/GA/Chi/IL-Pt/GCE) [5], but higher than the second antibody-multiwalled carbon nanotubes-cobalt phosphide/CEA/BSA/the first anti-body-gold nanoparticles modified GCE (Ab_2_-MWCNTs-CoP/CEA/BSA/Ab_1_-AuNPs/GCE) [51] and ferrocenecarboxaldehyde/Ru(bpy)_3_^2+^ doped SiO_2_ nanocomposite/complementary DNA/magnetic core-shell nanoparticles-modified AuE (Fc/Ru@SiO_2_/cDNA/Fe_3_O_4_@Au/AuE) [7]. In comparison with other electrodes that need the immobilization of a recognitive antibody/DNA or sandwich detection mode, the fabricated electrode exhibits advantages of simple fabrication, convenient operation and high sensitivity. 

rGO-Pd@Au-Thi-Ab_2_: reduced graphene oxide-Pd@Au nanocomposits-thionine-the second antibody; Ab_1_: the first antibody; Chi-Fc-Au: chitosan-ferrocenecarboxaldehyde-gold nanospheres; GCE: glass carbon electrode; MCH: 6-Mercapto-1-hexanol; TDNs: tetrahedral DNA nanostructures; AuE: gold electrode; Ru@SiO_2_: Ru(bpy)_3_^2+^ doped SiO_2_ nano-composite; Fe_3_O_4_@Au: magnetic core-shell nanoparticles; AuNPs: gold nanoparticles; Arg: L-arginine; Ti_3_C_2_-Mxene: ultrathin Ti_3_C_2_-MXene nanosheets; MWCNTs-CoP: multi-walled carbon nanotubes cobalt phosphide; Chi: chitosan; GA: glutaraldehyde; IL-Pt: GR-ionic liquid-platinum compound.

To investigate the detection selectivity of the sensor, the performance of the sensor for detecting different tumor markers, including alpha fetoprotein (AFP), prostate specific antigen (PSA), C-reactive protein (CRP) and breast cancer biomarker (CA153), is investigated. As shown in Figure 7c, no significant change of the peak current is observed after one of these substances is added. On the contrary, DPV peak current remarkably increases when CEA or the mixture containing CEA is incubated. This phenomenon indicates that the nanocomposite probe has high selectivity for CEA detection, which is attributed to the highly specific recognition between the aptamer and CEA.

### 2.6. Real Sample Analysis

To investigate the application potential of the developed homogeneous aptamer sensor, CEA detection in human serum is investigated using the standard addition method. As shown in Table 2, the detection recovery ranges from 98.1 to 105% with low relative standard deviation (RSD, no more than 3.7%), indicating high detection accuracy. Owing to the easy fabrication and simple operation, the developed homogeneous aptamer sensor demonstrates great potential in real sample analysis. 

## 3. Materials and Methods

### 3.1. Chemicals and Materials

Carcinoembryonic antigen (CEA), alpha fetoprotein (AFP), prostate specific antigen (PSA) and cancer antigen 153 (CA153) were purchased from Beijing KEY-BIO Biotech Co., Ltd. (Beijing, China). C-reactive protein (CRP) was obtained from Nanjing Okay Biotechnology Co., Ltd. (Nanjing, China). Amino-modified CEA aptamer (5′-ATACAGCTTCAATT-NH_2_-3′) was purchased from Sangon Biotechnology Co., Ltd. (Shanghai, China). Cetyltrimethylammonium bromide (CTAB), ethyl orthosilicate (TEOS), potassium ferricyanide (K_3_[Fe(CN)_6_]), potassium ferrocyanide (K_4_[Fe(CN)_6_]), sodium dihydrogen phosphate dihydrate (NaH_2_PO_4_·2H_2_O), disodium hydrogen phosphate dodecahydrate (Na_2_HPO_4_·12H_2_O), potassium chloride (KCl), glutaraldehyde (GA) and methylene blue trihydrate (MB) were obtained from Shanghai Aladdin Biochemical Technology Co., Ltd. (Shanghai, China). 3-Aminopropyltriethoxysilane (APTES) and potassium hydrogen phthalate (KHP) were purchased from Shanghai McLean Reagent Co., Ltd. (Shanghai, China). Ruthenium (III) chloride (Ru(NH_3_)_6_Cl_3_) and bovine serum albumin (BSA) were purchased from Sigma-Aldrich Co., Ltd. (Shanghai, China). Sodium nitrate (NaNO_3_) and sodium hydroxide (NaOH) were purchased from Hangzhou Gaojing Fine Chemical Co., Ltd. (Hangzhou, China). Ethanol and concentrated hydrochloric acid were purchased from Hangzhou Shuanglin Chemical Reagent Co., Ltd. (Hangzhou, China). Phosphate buffer solution (PBS) was prepared by mixing NaH_2_PO_4_·2H_2_O and Na_2_HPO_4_·12H_2_O in a certain proportion. All chemicals and reagents in this experiment were analytically pure and have not been further purified. All aqueous solution was prepared using ultrapure water (18.2 M Ω cm). Indium tin oxide (ITO) conductive glass electrode (square resistance < 17 Ω/square, thickness: 100 ± 20 nm) was purchased from Zhuhai Kaiwei Photoelectric Technology Co., Ltd. (Zhuhai, China). Before use, ITO electrode (0.5 cm × 0.5 cm) was immersed in NaOH (1 M) overnight. Then, the electrode was ultrasonically treated with acetone, ethanol and ultrapure water, successively. The clean electrode was finally dried with N_2_.

### 3.2. Measurements and Instrumentations

The surface and cross-sectional of VMSF was characterized using transmission electron microscopy (TEM, Hitachi HT7700, Tokyo, Japan) at 100 kV voltage. Cyclic voltammetry (CV) and differential pulse voltammetry (DPV) measurements were carried out on an Autolab (PGSTAT302N) electrochemical workstation (Metrohm, Herisau, Switzerland). UV-Vis spectra of GO were recorded using a UV-Vis spectrophotometer (UV-2600, Shimadzu, Tokyo, Japan). The functional groups of the nanomaterials were investigated using X-ray photoelectron spectroscopy (XPS) and Fourier transform infrared spectroscopy (FT-IR). XPS measurement was performed using a Mg Kα source excitation at 250 W and 14 kV (PHI5300, Perkin Elmer, Waltham, MA, USA). The FT-IR spectrum was obtained using the KBr plate method on a Vertex 70 spectrometer (Bruker, Billerica, MA, USA). Electrochemical measurements were performed at room temperature using the conventional three-electrode system. Specifically, bare ITO or modified ITO was the working electrode. Ag/AgCl and Pt electrodes are reference electrode and the auxiliary electrode, respectively. The step potential, pulse amplitude, pulse duration and inter pulse duration were 0.005 V, 0.025 V, 0.05 s and 0.2 s, respectively. Zeta potential was characterized on SZ-100V2 nanoparticle analyzer (Horibia, Tokyo, Japan).

### 3.3. Preparation of VMSF Modified ITO Electrode

VMSF was grown on the supporting ITO electrode using the EASA method [52,53,54]. Briefly, ethanol (20 mL), CTAB (4.35 mM), TEOS (3.050 mL) and NaNO_3_ (20 mL, 0.1 M, pH = 2.6) were mixed to prepare a precursor solution. Then, the mixture was pre-hydrolyzed at room temperature for 2.5 h with stirring. After a bare ITO electrode was immersed in the as-prepared precursor solution, a current density of −350 μA/cm^2^ was applied for 10 s. After VMSF growth, the electrode was quickly removed and thoroughly rinsed with ultrapure water. After aging at 120 °C overnight, the obtained electrode containing surfactant micelle (SM) was recorded as SM@VMSF/ITO. After this, SM was easily removed by immersing an SM@VMSF/ITO electrode in HCl-ethanol (0.1 M) solution for 10 min. An electrode modified with open nanochannel array was obtained (VMSF/ITO).

### 3.4. Preparation of Ru(NH_3_)_6_^3+^/Apt@GO Nanocomposite

Graphene oxide (GO) was prepared by Hummers method [55]. To prepare Ru(NH_3_)_6_^3+^/Apt@GO nanocomposite, Ru(NH_3_)_6_Cl_3_ (10 mM, 10 μL), GO (1 mg/mL, 110 μL) and CEA aptamer (10 μM, 100 μL) were added to PBS (0.01 M, pH = 7.4, 780 μL) and incubated for 3 h at 37 °C. Then, Ru(NH_3_)_6_^3+^/Apt@GO nanocomposite was obtained.

### 3.5. Electrochemical Detection of CEA and Real Sample Analysis

Different concentrations of CEA were incubated with Ru(NH_3_)_6_^3+^/Apt@GO nanocomposite probes at 37 °C for 1 h. The DPV curve of Ru(NH_3_)_6_^3+^ was detected using a VMSF/ITO electrode. For real sample analysis, human serum (healthy woman, provide by Shanxi Bethune Hospital) was diluted using PBS (0.01 M, pH = 7.4) by a factor of 50 before detection using the standard addition method.

## 4. Conclusions

In summary, a homogeneous electrochemical aptamer sensor is fabricated based on a 2D nanocomposite probe and nanochannel modified electrode, which can realize sensitive detection of CEA. Vertically ordered mesoporous silica-nanochannel film is easily grown on the surface of the supporting ITO electrode. The as-prepared nanochannel-modified electrode exhibits the advantages of low cost and convenient production. A 2D nanocomposite probe can be easily prepared using a graphene oxide matrix through simple π-π interaction and electrostatic adsorption between recognitive aptamer and electrochemical signal molecules, Ru(NH_3_)_6_^3+^. In the presence of CEA, the specific binding between aptamer and CEA leads to the detach of Ru(NH_3_)_6_^3+^ to enter the solution. As VMSF nanochannels exhibit electrostatic enrichment towards Ru(NH_3_)_6_^3+^ and size exclusion towards 2D material, the solution-based Ru(NH_3_)_6_^3+^ can be sensitively detected with low background signal, leading to the sensitive detection of CEA from 60 fg/mL to 100 ng/mL with a low limit of detection (14 fg/mL). The fabricated sensor also displays high selectivity. The constructed homogeneous aptamer sensor has a simple fabrication process which does not require the fixation of recognition ligands on the electrode surface and pre-separation before detection, indicating great potential in the rapid and convenient detection of tumor biomarkers.

## Figures and Tables

**Table 1:** Comparison between CEA detection performance using different electrodes.

Electrode	Detection Method	Linear Range (ng/mL)	LOD (fg/mL)	References
rGO-Pd@Au-Thi-Ab_2_/CEA/ BSA/Ab_1_/Chi-Fc-Au/GCE	EC	10^−4^/200	149	[8]
BSA/MCH/TDNs/AuE	EC	10^−3^/30	45.7	[50]
Ab_2_-MWCNTs-CoP/CEA/ BSA/Ab_1_-AuNPs/GCE	EC	10^−4^/10	12	[51]
AuNPs-Ru-Arg@NH_2_-Ti_3_C_2_-MXene	ECL	0.01/200	1500	[6]
Fc/Ru@SiO_2_/cDNA/Fe_3_O_4_@Au/AuE	ECL	1 × 10^−5^/10	3.5	[7]
BSA/Ab_1_/GA/Chi/IL-Pt/GCE	ECL	10^−5^/100	34.6	[5]
VMSF/ITO	EC	6 × 10^−5^/100	14	This work

**Table 2:** Determination of CEA in human serum.

Sample	Added ^b^ (ng/mL)	Found (ng/mL)	Recovery (%)	RSD (%, n = 3)
Serum ^a^	0.0100	0.0102	102	1.9
	1.00	1.05	105	3.7
	100	98.1	98.1	1.6

^a^ Serum sample is diluted by a factor of 50 using PBS (0.01 M, pH 7.4). ^b^ The concentration of CEA indicated in the table is obtained after the dilution.

## Data Availability

The data presented in this study are available on request from the corresponding author.
